# Ahmedabad tolerance induction protocol and chronic renal allograft dysfunction: pathologic observations and clinical implications

**DOI:** 10.1186/1746-1596-4-4

**Published:** 2009-01-30

**Authors:** Rashmi D Patel, Aruna V Vanikar, Feroz A Aziz, Pankaj R Shah, Hargovind L Trivedi

**Affiliations:** 1 Department of Pathology, Laboratory Medicine, Transfusion Services and Immunohematology, G.R. Doshi and K.M. Mehta Institute of Kidney diseases And Research Centre and Dr. H.L. Trivedi Institute of Transplantation Sciences, Ahmedabad, India; 2 Department of Nephrology and Transplantation Medicine, G.R. Doshi and K.M. Mehta Institute of Kidney diseases And Research Centre and Dr. H.L. Trivedi Institute of Transplantation Sciences, Ahmedabad, India

## Abstract

**Background:**

Chronic Renal Allograft Dysfunction (CRAD) is responsible for a large number of graft failures. We have abrogated acute T-cell rejections using Ahmedabad Tolerance Induction Protocol (ATIP) with hematopoietic stem cell transplantation (HSCT) under non-myeloablative conditioning pre-transplant. However B-cell mediated rejections and CRAD continue to haunt us. We carried out retrospective analysis of renal allograft biopsies performed in the last 4 years to evaluate the effect of ATIP on CRAD.

**Materials and methods:**

Biopsies diagnosed as per modified Banff criteria belonged to 2 groups: ATIP under low dose immunosuppression of cyclosporine/Azathioprine/Mycofenolate mofetil+ Prednisolone, subjected to donor leucocyte transfusion, anti-T/B cell antibodies, low dose target specific irradiation, cyclophosphamide, cyclosporin followed by HSCT pre-transplant; controls who opted out of ATIP were transplanted under standard triple drug immunosuppression. Demographics of both groups were comparable.

**Results:**

Incidence of chronic changes was higher in controls (17.5%) vs. 10.98% in ATIP over a mean follow up of 151.9 months in the former and 130.9 months in the latter. Proteinuria and hypertension were higher in controls (48.4%) vs. ATIP (32.7%) with chronic transplant glomerulopathy, focal global sclerosis in 67.7% in controls vs. 46.7% in ATIP, acute on chronic T/B cell rejection in 51.6% controls vs. 28.1% ATIP, with peritubular capillary C4d deposits in 19.4% controls vs. 1.9% ATIP biopsies. Acute on chronic calcineurin inhibitor toxicity was higher in ATIP (71.9%) vs. 48.4% in controls.

**Conclusion:**

Chronic immune injury was less with ATIP vs controls as compared to a higher incidence of chronic calcineurin inhibitor toxicity in the former.

## Background

Significant improvement in 1 year renal allograft and patient survival rates have been achieved over the last 10 years as a result of newer immunosuppressive regimes and the increasing use of living donors for transplantation. However long term graft function and survival rates remain less than optimal. Up to 50% grafts are lost to chronic renal allograft dysfunction (CRAD). [1] We have been modifying Ahmedabad Tolerance Induction Protocol (ATIP) to achieve transplantation tolerance in our renal allograft recipients since 1998. [2] We have effectively abrogated acute T-cell mediated rejections however we have not been able to address B-cell mediated rejections and CRAD. The present study aims to evaluate the effect of ATIP on CRAD in our living-related renal allograft recipients.

## Materials and methods

This was a retrospective study of renal allograft biopsies performed at our center from January 2004 to December 2007. Out of 1699 patients transplanted from January 1998 to October 2007, 727/1171 (62.1%) ATIP and 312/528 (59.1%) controls with functioning grafts are on follow-up.

ATIP comprised of administration of donor leucocyte transfusions, target specific irradiation to sub-diaphragmatic lymph nodes, spleen, part of pelvic bones and lumbar vertebrae (200 CgY × 5 days), anti-T cell antibody, 1.5 mg/kg BW and anti-B cell antibody, 6 mg/kg BW intravenously respectively followed by unmodified cytokine stimulated donor derived bone marrow (BM) administration intraportally fortified with cultured BM derived hematopoietic stem cells, human adipose tissue derived mesenchymal stem cells and peripheral blood stem cells to recipients. Renal transplantation was performed following negative lymphocytotoxicity cross-matching (LCM) (serology).

Patients belonging to the control group were transplanted following negative LCM reports. Recipient maintenance immunosuppression of ATIP comprised of Cyclosporin, 1.5 ± 0.5 mg/kgBW/day or Azathioprine, 2 mg/kg BW/day or Mycofenolate mofetil, 1 gm/day and Prednisolone, 7.5 ± 2.5 mg/day. Control group patients were on standard triple drug immunosuppression of Cyclosporin, 4.5 ± 1.5 mg/kgBW/day/Azathioprine, 2 mg/kg BW/day/Mycofenolate mofetil 2 gm/day and Prednisolone, 15 ± 5 mg/day.

Demographics of both groups were comparable.

Biopsies were performed whenever there was a sustained rise of > 10% baseline serum creatinine (SCr) for 10 days. Biopsies with chronic changes were included in this study and diagnosed as per modified Banff criteria. [3,4] Morphological evaluation was carried out using hematoxylin and eosin, periodic acid Schiff, Jones' silver methaneamine and Gomori's trichrome stains on 3 μm paraffin sections. C4d antibodies were tested using polyvalent anti-human C4d antiserum (Biomedica Gruppe, Beckman Coulter, Germany) by both techniques, immunofluorescence (IF) and immunohistochemistry (IHC) (using their working protocol). Membranous nephropathy slides were used as outside positive controls.

Chronic changes were classified into 2 patterns; those with presence of stripped interstitial fibrosis, subintimal diffuse/segmental arteriolar hyalinosis, negative C4d deposits, absence of transplant glomerulopathies and presence of periglomerular fibrosis were labeled as chronic changes attributed to calcineurin inhibitor (CNI) toxicity. The other group with variable fibrosis (and absence of stripped fibrosis) with superadded lymphoplasmacytic and/or neutrophilic infiltrates, with/without chronic transplant glomerulopathy, C4d deposits (diffuse/focal) across peritubular capillary membranes (by IF or IHC) and chronic transplant vasculopathy were attributed to chronic B-cell (antibody) mediated rejections with/without ongoing acute injuries. A subset of these biopsies was also subjected to CD20 stain by IHC to confirm B-cell involvement.

## Results

Out of the 1151 graft biopsies performed between January 2004 to December 2007, 135 (11.8%) biopsies with chronic changes were reviewed. In total there were 107 out of 974 (10.98%) biopsies belonging to ATIP group, and 31 out of 177 (17.5%) biopsies from the control group showing chronic changes. Hypertension (with BP > 130/90 mmHg) was noted in 71 (66.4%) the ATIP patients and 13 (41.9%) controls. Proteinuria (with 24 hours urinary protein leak of > 800 mg) was recorded in 46/107 (43%) ATIP and 25/31 (80.6%) controls.

**Figure 1:**
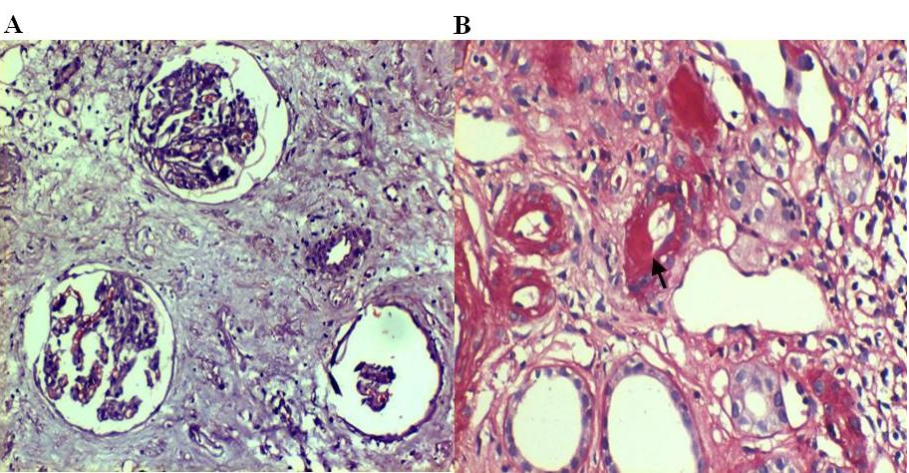
**a **Atubular glomeruli surrounded by interstitial fibrosis in chronic allograft dysfunction associated with chronic Calcineurin inhibitor toxicity, Hematoxylin and eosin stain, × 100. **b **Small caliber artery showing subintimal segmental hyalinosis (arrow head) as part of chronic CNI toxicity, Periodic Acid Schiff stain, × 200.

**Figure 2:**
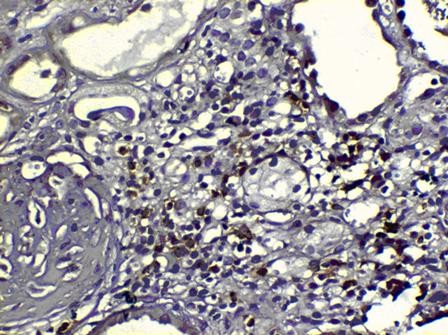
**CD20 stain showing B-cells (arrow) in ongoing chronic rejection, × 200**.

In ATIP, 77/107 (71.9%) biopsies and in controls, 15/31 (48.4%) biopsies qualified for chronic changes attributed to CNI toxicity (figure 1a, b). Chronic changes due to immune mediated injuries were noted in 30/107 (28.1%) ATIP group and 16/31 (51.6%) controls (figure 2).

CNI toxicity in ATIP group was noted in 63 males and 14 females with mean age of 34 years (range: 15–63 years) with a mean follow-up of 4.09 years (range: 0.66–8.98 years).

Their mean SCr was 3 mg % (range: 0.8–12.9 mg %).

CNI toxicity in the control group was noted in 10 males and 5 females with a mean age of 34 years (range: 17–52 years) with a mean follow-up of 5 years (range: 0.45–9.21 years). Their mean SCr was 3 mg % (range: 1.6–4.13 mg %).

In the ATIP group, chronic ongoing immune injury was noted in 24 males and 6 females with a mean age of 33.6 years (range: 12–56 years) with a mean follow-up of 4.53 years (range: 0.6–9.11 years) and their mean SCr was 3.02 mg% (range: 1.2–8.3 mg %).

In control group, chronic ongoing immune injury was noted in 11 males and 5 females with mean age of 32.8 years (range: 11–67 years) with mean follow-up of 4.63 years (range: 0.65–8.24 years) and their mean SCr was 3.8 mg% (range: 1.6–8.5 mg %). The results are mentioned in table 1.

**Table 1:** Chronic changes noted in renal allograft biopsies of 2 groups, Ahmedabad Tolerance Induction Protocol (ATIP) vs. controls:

**Biopsies**	**ATIP**	**Controls**
**(n = 1151)**	**(n = 974)**	**(n = 177)**


Chronic changes (n = 135)	107 (10.98%)	31 (17.5%)

Mean follow-up (months) (range)	130.9 (23–277)	151.9 (13.9–280)

Male: female	87:20:00	21:10

Hypertension (BP > 130/90 mm Hg)	71 (66.4%)	13 (41.9%)

Proteinuria (24 hrs urine albumin > 800 mg)	46 (43%)	25(80.6%)

HISTOPATHOLOGY		

Interstitial fibrosis+ tubular atrophy	107 (100%)	31 (100%)

Focal global sclerosis	50 (46.7%)	21 (67.7%)

Chronic transplant glomerulopathy	35 (32.7%)	15 (48.4%)

C4d deposits	2(1.9%)	6 (19.4%)

Acute on chronic calcineurin inhibitor toxicity	77 (71.9%)	15 (48.4%)

Acute on chronic T/B cell rejection	30 (28.1%)	16 (51.6%)

## Discussion

Renal transplantation is the best therapeutic option for patients with end-stage renal disease. However acute rejections, infections, recurrence and drug toxicity are the major limitations for graft/patient loss in the first year of transplantation and chronic rejections are added to the list to lead to graft and patient loss later on. [5-7]

CRAD has been defined as a progressive renal dysfunction, independent of acute rejection, drug toxicity and recurrent/de novo nephropathy with histological changes of vascular intimal hyperplasia, tubular atrophy, interstitial fibrosis and chronic transplant glomerulopathy. Injuries associated with CNI appear to be very common. The pathophysiology of CRAD is complex and multi-factorial. Both allo-antigen dependent factors like acute rejections, HLA matching, ABO incompatibility, donor-specific antibodies, inadequate immunosuppression, and allo-antigen independent factors like donor age, living-related/deceased donor, ischemia reperfusion injury, CNI/viral related injuries, hypertension, hyperlipidemia, diabetes mellitus, etc. are involved in its causation. The 8^th ^consensus meeting for Banff classification in 2005 decided to eliminate the term chronic allograft nephropathy (CAN) and replace it with chronic alloimmune versus non-immune injury. [3,4]

We have been able to effectively address the problems of acute T-cell mediated rejections and infections in the first year of transplantation with continuous modifications of ATIP whereby we have achieved prope tolerance. [8-10] However we have not been able to address to B-cell antibody mediated rejections and CRAD. We have seen that our patients present with a standard pattern of slow decrease in glomerular filtration rate (GFR) reflected by progressive increase in serum creatinine (SCr), hypertension in about 66.4% of ATIP patients and 41.9% controls, with/without proteinuria within the nephrotic range.

ABO antibodies were not the confounding factors in our patients. We believe that those anti-HLA and non-HLA antibodies that could not be abrogated by the ATIP group were responsible for the chronic changes that would eventually lead to graft loss.

We hypothesize that in ATIP group, exhaustion of the T-cell repertoire leads to free availability of endothelial sites to the drug and hence it eventually causes chronic graft attrition.

The two groups in this study are unequal in size since most of our patients opt for transplantation with ATIP due to its overwhelming benefits. The salient features noted in this study are that beyond one year of transplantation, long term effects of ATIP are:

1. markedly reduced requirement of immunosuppression

2. Even lower CNI dose leads to chronic changes eventually causing graft attrition as noted in 71.9% ATIP biopsies vs. 48.4% controls.

3. Partial protection from immune injury as observed in 28.1% ATIP biopsies vs. 51.6% controls.

Further studies are ongoing to study donor specific antibodies, non-specific antibodies and the role of MICA antibodies on graft loss.

## Conclusion

Retrospective evaluation of renal allograft biopsies shows that transplantation using ATIP yields lesser incidence of chronic immune injuries as compared to controls and have a higher incidence of chronic CNI toxicity even at lower doses.

## Abbreviations

ATIP: Ahmedabad Tolerance Induction Protocol; CNI: calcineurin inhibitor; CRAD: chronic renal allograft dysfunction; LCM: lymphocytotoxicity cross-matching.

## Competing interests

The authors declare that they have no competing interests.

## Authors' contributions

RP and AV have carried out the all the laboratory work, V has designed the study. AF and PRS have carried out all the clinical work and HLT is the supervisor of the entire project and has also personally monitored every patient.
